# PPAR-γ Activation Increases Insulin Secretion through the Up-regulation of the Free Fatty Acid Receptor GPR40 in Pancreatic β-Cells

**DOI:** 10.1371/journal.pone.0050128

**Published:** 2013-01-23

**Authors:** Hyo-Sup Kim, You-Cheol Hwang, Seung-Hoi Koo, Kyong Soo Park, Myung-Shik Lee, Kwang-Won Kim, Moon-Kyu Lee

**Affiliations:** 1 Division of Endocrinology and Metabolism, Samsung Biomedical Research Institute, Sungkyunkwan University School of Medicine, Seoul, Korea; 2 Division of Endocrinology and Metabolism, Department of Medicine, Kyung Hee University Hospital at Gangdong, Kyung Hee University School of Medicine, Seoul, Korea; 3 Department of Molecular Cell Biology, Sungkyunkwan University School of Medicine, Seoul, Korea; 4 Department of Internal Medicine, Seoul National University College of Medicine, Seoul, Korea; 5 Division of Endocrinology and Metabolism, Department of Medicine, Samsung Medical Center, Sungkyunkwan University School of Medicine, Seoul, Korea; Universita Magna-Graecia di Catanzaro, Italy

## Abstract

**Background:**

It has been reported that peroxisome proliferator-activated receptor (PPAR)-γ and their synthetic ligands have direct effects on pancreatic β-cells. We investigated whether PPAR-γ activation stimulates insulin secretion through the up-regulation of GPR40 in pancreatic β-cells.

**Methods:**

Rat insulinoma INS-1 cells and primary rat islets were treated with rosiglitazone (RGZ) and/or adenoviral PPAR-γ overexpression. OLETF rats were treated with RGZ.

**Results:**

PPAR-γ activation with RGZ and/or adenoviral PPAR-γ overexpression increased free fatty acid (FFA) receptor GPR40 expression, and increased insulin secretion and intracellular calcium mobilization, and was blocked by the PLC inhibitors, GPR40 RNA interference, and GLUT2 RNA interference. As a downstream signaling pathway of intracellular calcium mobilization, the phosphorylated levels of CaMKII and CREB, and the downstream IRS-2 and phospho-Akt were significantly increased. Despite of insulin receptor RNA interference, the levels of IRS-2 and phospho-Akt was still maintained with PPAR-γ activation. In addition, the β-cell specific gene expression, including Pdx-1 and FoxA2, increased in a GPR40- and GLUT2-dependent manner. The levels of GPR40, phosphorylated CaMKII and CREB, and β-cell specific genes induced by RGZ were blocked by GW9662, a PPAR-γ antagonist. Finally, PPAR-γ activation up-regulated β-cell gene expressions through FoxO1 nuclear exclusion, independent of the insulin signaling pathway. Based on immunohistochemical staining, the GLUT2, IRS-2, Pdx-1, and GPR40 were more strongly expressed in islets from RGZ-treated OLETF rats compared to control islets.

**Conclusion:**

These observations suggest that PPAR-γ activation with RGZ and/or adenoviral overexpression increased intracellular calcium mobilization, insulin secretion, and β-cell gene expression through GPR40 and GLUT2 gene up-regulation.

## Introduction

Peroxisome proliferator-activated receptor (PPAR)-γ is a member of the nuclear receptor family that plays a crucial role in lipid and glucose homeostasis. It is well known that thiazolidinediones (TZDs), synthetic ligands for PPAR-γ, exert their glucose-lowering effects principally via improving peripheral insulin sensitivity [1], [2]. However, some studies indicate that TZDs have direct effects on glucose-stimulated insulin secretion (GSIS) and pancreatic β-cell gene expression [3]–[10]. Furthermore, it has been reported that TZDs protect β-cells from the pro-inflammatory cytokines such as interleukin-1β and interferon-γ [11], [12], human islet amyloid polypeptide (h-IAPP) [13], [14], free fatty acid (FFA) toxicity [15]–[18], and endoplasmic reticulum (ER) stress [19].

G-protein-coupled transmembrane receptor 40 (GPR 40) is a membrane-bound FFA receptor mainly expressed in the brain and pancreatic β-cells [20]–[22]. Accumulating evidence indicates that GPR40 mediates the majority of both acute and chronic effects of FFAs on insulin secretion, including the amplification of GSIS [21], [23]–[29], and the receptor has been suggested to be involved in the control of cell proliferation via extracellular signal-related kinase (ERK), phosphoinositide 3-kinase (PI3K), and PKB signaling pathways [30]. GPR40 is also expressed in enteroendocrine cells, including cells expressing incretin hormones, glucagon like peptide-1 (GLP-1) and glucose-dependent insulinotrophic peptide (GIP), and it modulates FFA-stimulated insulin secretion not only from pancreatic β-cells, but also through the regulation of incretin hormones [31].

Recently, it was reported that TZDs may preferentially activate the GPR40 receptor, resulting in Ca^2+^ mobilization from thapsigargin-sensitive intracellular stores that would induce cell growth, whereas the endogenous PPAR-γ ligand, 15-deoxy-Δ^12,14^-prostaglandin J_2_ (15 d-PGJ_2_), did not induce any Ca^2+^ signal and inhibited cell growth in nonmalignant human bronchial epithelial cells [22], [32]. Taken together, TZDs increase intracellular Ca^2+^ from the ER through GPR40 receptor activation in a PPAR-γ-independent manner.

In this context, we investigated whether PPAR-γ activation stimulates insulin secretion through the up-regulation of GPR40 in INS-1 cells. We also explored the GPR40 downstream signaling pathways involved in the role of PPAR-γ activation in pancreatic β-cells.

## Methods

### Materials

Rosiglitazone (RGZ) was obtained from Alexis (Leusen, Switzerland). The U-73122 and nifedipine were purchased from Calbiochem (Merk, Nottingham, UK). All other reagents were purchased from Sigma-Aldrich (St. Louis, MO) unless noted.

### Cell culture

Rat insulinoma INS-1 cells were kindly provided by Dr. P. Maechler (Geneva, Switzerland) [33]. INS-1 cells were maintained in RPMI 1640 medium containing 11 mM glucose supplemented with 10 mM HEPES, 10% heat-inactivated fetal bovine serum (FBS), 2 mM L-glutamine, 1 mM sodium pyruvate, 50 µM β-mercaptoethanol, 100 IU/ml penicillin, and 100 µg/ml of streptomycin in a humidified atmosphere (5% CO_2_, 95% air). In the starvation condition, RPMI-1640 media containing 2% bovine serum albumin (BSA) was used.

### Islet isolation

Islets were isolated from the pancreas of male Sprague Dawley rats (Orientbio, Seongnam, Gyeonggi-do, Korea) by distending the pancreatic duct with a mixture of collagenase (Roche). After the digestion at 37°C, the islets were separated on a discontinuous histopaque density gradient (Histopaque 1077; Sigma) and further purified by handpicking. Handpicked islets were cultured in sponge (Spongostan®; Johnson & Johnson, Denmark) with RPMI 1640 medium. All the procedures were approved by the Institutional Animal Care and Use Committee at Samsung Biomedical Research Institute.

### Ca^2+^ detection assay

After treatment with RGZ and/or other chemicals for 24 h, cells were stimulated with 16.7 mM glucose in Krebs–Ringer bicarbonate buffer (KRBB) solution for 1 h. After glucose stimulation, cells were treated with 2 µM Fluo-4 (Molecular Probes, Eugene, OR) in calcium and glucose-free KRBB solution for 30 min in the incubator with light protection, and then washed three times with calcium and glucose-free KRBB solution. Cells were observed under a fluorescence microscope (Olympus, Tokyo, Japan).

### Measurement of insulin release

The medium was replaced with defined serum-free medium containing RGZ, and other inhibitors at the designated concentrations. After 24 h of treatment, cells were washed with Krebs-Ringer-bicarbonate-HEPES (KRBH) buffer and incubated for an additional 60 min in 1 ml of KRBH buffer containing 5.6 or 16.7 mM of glucose. Insulin secretion was normalized for cell number by measuring total protein in each experiment with the Bradford assay, and was determined using a rat insulin ELISA kit (Mercodia, Uppsala, Sweden). FFAs including linoleic, oleic and palmitic acid in 0.01 M NaOH was incubated at 60°C for 30 min, and then FFAs were complexed with 5% FFA-free BSA in PBS. The FFA/BSA conjugates were used to treat INS-1 cells.

### Perifusion for insulin secretion

After GPR40 RNAi transfection, secreted insulin was measured in a perifusion system (Cellex biosciences, inc., Minneapolis, MN). 1×10^5^ INS-1 cells were cultured in small chambers on Millicell culture inserts. INS-1 cells were perifused in 3.3 mM glucose KRBB media for 30 mins at flow rate of 1 ml/min at 37°C chamber. Glucose concentrations were modified at 16.7 mM concentration. Fractions were collected at 1 min intervals during the 1^st^ peak insulin secretion and then collected at 2 min intervals. Collected fractions were stored at −20°C before performing insulin ELISA.

### Western blotting

Thirty micrograms of proteins per lane were electrophoresed on 10% polyacrylamide gels and electroblotted onto nitrocellulose membranes (Millipore, Bedford, MA). Blots were blocked with 5% skim milk in Tris-buffered saline containing 0.1% Tween-20 (T-TBS) for 40 min and incubated overnight at 4°C with the primary antibodies. Nuclear and cytoplasmic extraction to determine FoxO1 cellular localization was made using NE-PER nuclear and cytoplasmic extraction reagents (Pierce, Rockford, IL) according to the manufacturer instructions. Blots were developed by enhanced chemiluminescence using a standard kit (Amersham Pharmacia Biotech, Piscataway, NJ). Western blot band density was analyzed using a GS-800 calibrated densitometer (Bio-Rad, Hercules, CA, USA).

### Adenovirus infection & RNAi transfection

Adenovirus containing human PPAR-γ1 complementary DNA was presented by Dr. KS Park (Seoul, Korea) and adenovirus containing rat FoxO1 shRNA was provided by Dr. SH Koo (Seoul, Korea). Adenoviruses were applied to INS-1 cells and islets at 3.0×10^6^ pfu/ml for PPAR-γ overexpression and 1.0×10^6^ pfu/ml for FoxO1 suppression. The efficacy of infection for varying viral loads was determined by LacZ staining for Ad-PPAR-γ and green fluorescent protein (GFP) observation for Ad-FoxO1-GFP (Figure S1A). Insulin receptor, GLUT2, and GPR40 sequence-specific silencing was performed with 100 pM/µl of RNAi (Bioneer, Daejon, Korea) using HiPerFect transfection reagent (Invitrogen, San Francisco, CA), according to the manufacturer's instructions. Suppression of target proteins was measured with Western blots (Fig. S1B).

### Hoechst 33258 staining

Hoechst-33258 (bisbenzimide trihydrochloride, HO258; Calbiochem, La Jolla, CA) was used to detect cell apoptosis. Nuclei were visualized under a fluorescence microscope (Olympus, Tokyo, Japan) at 348 nm (excitation) and 480 nm (emission).

### Oral glucose tolerance test (OGTT)

All procedures were performed in accordance with the recommendations in the Guide for the Care and Use of Laboratory Animals of the National Institutes of Health. The protocol was approved by the Committee on the Ethics of Animal Experiments of the Samsung Biomedical Research Institute (SBRI), Sungkyunkwan University School of Medicine (Permit Number: H-B0-043). Male OLETF rats and their diabetes-resistant counterparts, LETO rats, were supplied by Tokushima Research Institute (Otsuka Pharmaceutical, Tokushima, Japan) at 8 weeks of age. At an age of 10 weeks, OLETF rats were randomly assigned to the RGZ treatment or control group, and 3 mg/kg of RGZ was given by mouth through gavage. After 14 weeks of RGZ treatment (24 weeks of age), OGTT was performed. Glucose (2 g/kg) solution was given orally, and serum glucose levels were measured before (0 min) and 30, 60, 90 and 120 min after glucose loading.

### Hyperinsulinemic euglycemic clamp

One week before the experiment, animals were chronically cannulated in the jugular vein for infusion of glucose and insulin, and in the carotid artery for sampling. Cannulae were tunneled subcutaneously and exteriorized at the back of the neck. Animals were allowed 7 days to recover from surgery and to regain body weight. RGZ-treated rats continued to receive a RGZ-containing chow during the recovery period. Food was withdrawn 6 h before the hyperinsulinemic-euglycemic clamping. After 14 weeks of treatment (24 weeks of age), hyperinsulinemic-euglycemic clamp studies were performed. Basal samples were drawn at −30 and 0 min. Animals were exposed to a hyperinsulinemic clamp in which the insulin infusion rates were 4 (sub-maximum) and 40 (maximum) mU/kg/min. Blood was sampled for measurement of plasma glucose concentration every 5 min and for insulin levels every 20–30 min. Plasma glucose concentrations were immediately determined, and the remaining sample was stored at −20°C for later assay.

### Immunohistochemistry

At 24 weeks of age, OLETF rats were sacrificed by an intraperitoneal injection of sodium pentobarbital (45 mg/kg), and all efforts were made to minimize suffering. Specimens were obtained from the distal end of the pancreas. The tissue was fixed in 4% paraformaldehyde, dehydrated, embedded in paraffin, and sequentially sectioned.

### Statistics

The data are presented as the means ± SD. The Mann-Whitney tests were performed to compare differences between two independent groups. One-way ANOVA with post-hoc analyses were used to compare differences between several groups. *P* values <0.05 were considered statistically significant (PRISM; Graphpad Software Corp., San Diego, CA).

## Results

### Effects of PPAR-γ activation on insulin secretion and intracellular calcium mobilization

**Figure 1:**
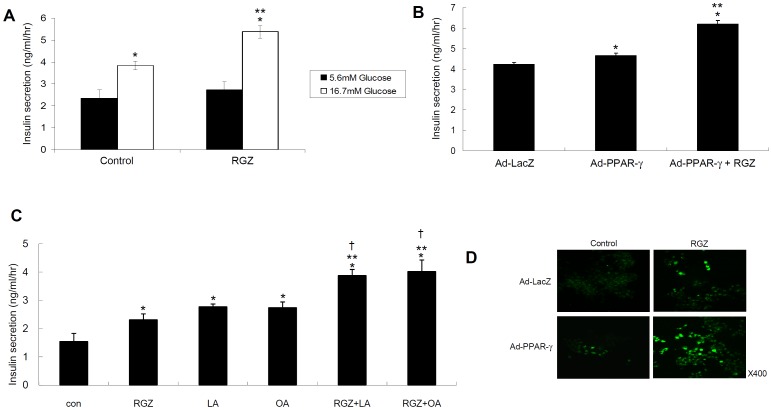
Effects of PPAR-γ activation on insulin secretion and intracellular calcium mobilization. (A) INS-1 cells were treated with 10 µM RGZ for 24 h, and incubated for an additional 1 h in 5.6 or 16.7 mM of glucose conditions. Insulin was determined using a rat insulin ELISA kit (*n* = 4, ^*^ *P*<0.01 vs. 5.6 mM glucose; ^**^ *P*<0.01 vs. control with 16.7 mM glucose). (B) INS-1 cells were infected with adenovirus containing human PPARγ1 complementary DNA or control virus. Insulin secretion was determined in 16.7 mM of glucose condition (*n* = 4, ^*^ *P*<0.05 vs. control virus treated cells; ^**^ *P*<0.01 vs. adenoviral PPAR-γ overexpression). (C) INS-1 cells were treated with 10 µM RGZ for 24 h, and then 10 µM linoleic acid (LA) or oleic acid (OA) were added for 30 minutes (*n* = 4, ^*^ *P*<0.01 vs. control; ^**^ *P*<0.01 vs. RGZ treatment; and **^†^** *P*<0.01 vs. LA or OA treatment). (D) After treatment with RGZ and/or adenoviral PPARγ overexpression for 24 h, cells were stimulated with 16.7 mM glucose for 1 h, and then treated with 2 µM Fluo-4 for 30 min in the incubator with light protection.

Figure 1A showed that 24 h of treatment with 10 µM RGZ increases GSIS in high (16.7 mM) glucose conditions (*P*<0.01 vs. control), and adenoviral PPAR-γ overexpression also exhibits increased insulin secretion on stimulation with 16.7 mM glucose. Furthermore, co-treatment with RGZ and PPAR-γ overexpression augmented the insulin secretion compared with PPAR-γ overexpression alone. However, 10 µM RGZ treatment did not show any increment in insulin secretion in basal (5.6 mM) glucose conditions (Fig. 1A, B). The stimulatory activities of 10 µM oleic or linoleic acids for 30 minutes on insulin secretion were detected more strongly when pretreated with 10 µM RGZ, indicating that FFAs amplify RGZ-stimulated insulin secretion from INS-1 cells (Fig. 1C). In accordance with the results of insulin secretion, Fluo-4 stained intracellular calcium was increased with RGZ treatment or PPAR-γ overexpression compared with control, and co-treatment with RGZ and PPAR-γ overexpression showed a synergetic effect on intracellular calcium mobilization (Fig. 1D).

### RGZ induces intracellular calcium mobilization from extra- and intra-cellular sources

**Figure 2:**
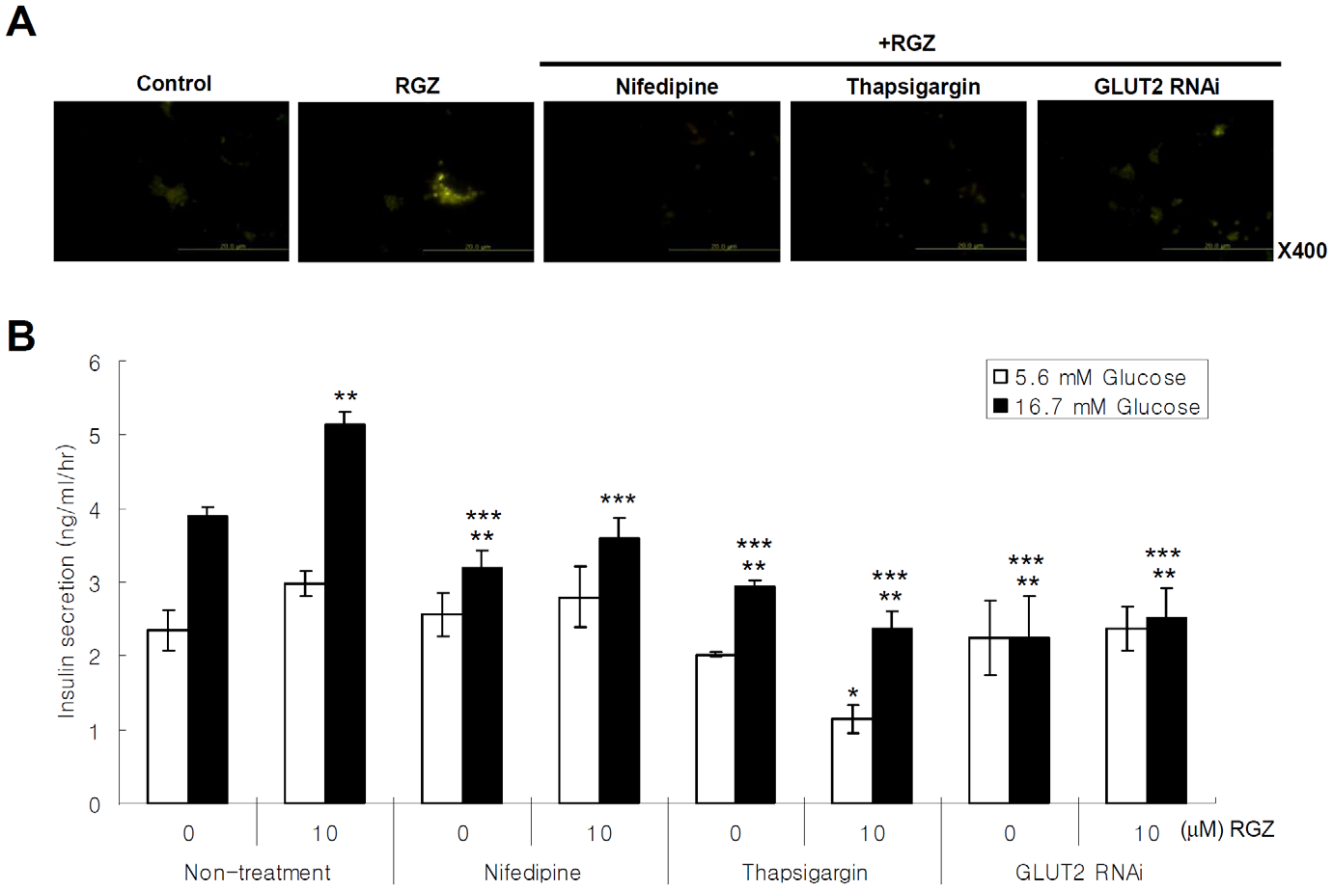
RGZ induces intracellular calcium mobilization from extra- and intra-cellular sources. (A) Effects of nifedipine (10 mM), thapsigargin (0.1 µM), and GLUT2 sequence-specific silencing with RNAi on 10 µM RGZ-induced intracellular calcium mobilization and (B) insulin secretion (ANOVA within same glucose conditions: *n* = 4, ^*^ *P*<0.01 vs. RGZ treatment with 5.6 mM glucose; ^**^ *P*<0.05 vs. non-treatment with 16.7 mM glucose; and ^***^ *P*<0.01 vs. RGZ treatment with 16.7 mM glucose) (C) Immunoblot for GLUT2 expression with 10 µM RGZ treatment and/or adenoviral PPAR-γ overexpression.

To determine the sources of increased intracellular calcium in response to PPAR-γ activation, we used 10 mM nifedipine to block the calcium influx from the extracellular source. Compared with RGZ treatment alone, co-treatment with RGZ and nifedipine decreased the Fluo-4 stained calcium (Fig. 2A). As is the case of nifedipine, 0.1 µM thapsigargin treatment blocked the RGZ-induced intracellular calcium mobilization (Fig. 2A). Taken together, PPAR-γ activation increases intracellular calcium from both extra- and intra-cellular sources and as expected, RGZ-induced insulin secretion was decreased with nifedipine and thapsigargin treatment under high glucose conditions (Fig. 2B).

RGZ treatment and PPAR-γ overexpression increased GLUT2 expression, and co-treatment with RGZ and PPAR-γ overexpression showed a synergetic effect on GLUT2 expression (Fig. 2C). In addition, GLUT2-specific RNAi blocked a RGZ-induced intracellular calcium mobilization and GSIS (Fig. 2A, B).

### PPAR-γ activation increases intracellular calcium concentration and insulin secretion through GPR40 gene up-regulation

**Figure 3:**
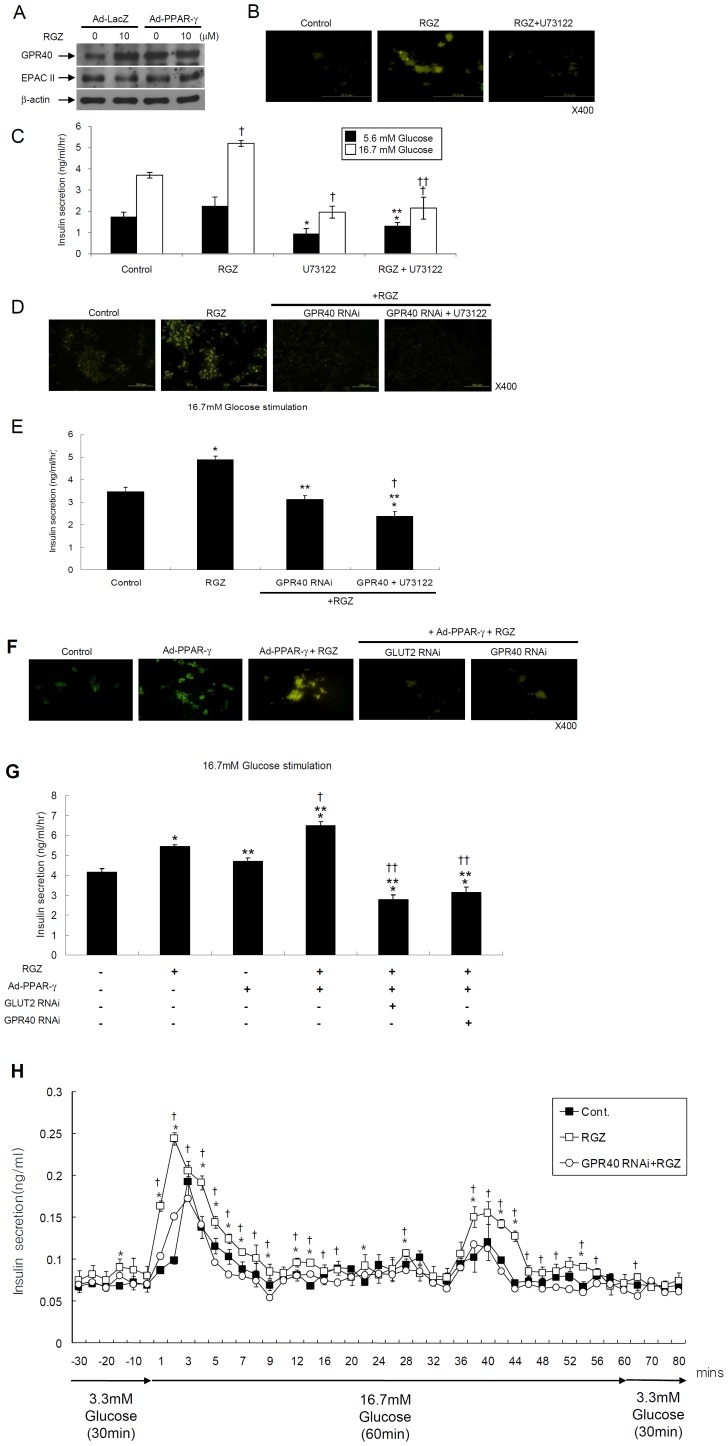
PPAR-γ activation increases intracellular calcium concentration and insulin secretion through GPR40 gene up-regulation. (A) Immunoblot for GPR40 and EPAC II expression with 10 µM RGZ treatment and/or adenoviral PPAR-γ overexpression. (B) Effects of PLC inhibitor, U-73122 (20 µM), on RGZ-induced intracellular calcium mobilization and (C) insulin secretion (ANOVA within same glucose conditions: *n* = 4, ^*^ *P*<0.01 vs. control RGZ treatment with 5.6 mM glucose; ^**^ *P*<0.01 vs. RGZ or U-73122 treatment with 5.6 mM glucose; ^†^ *P*<0.01 vs. control with 16.7 mM glucose; and ^††^ *P*<0.01 vs. RGZ or U-73122 treatment with 16.7 mM glucose). (D) Effects of GPR40 sequence-specific silencing with RNAi and/or U-73122 (20 µM), on RGZ-induced intracellular calcium mobilization and (E) insulin secretion (*n* = 4, ^*^ *P*<0.01 vs. control; ^**^ *P*<0.01 vs. RGZ treatment; and **^†^** *P*<0.01 vs. RGZ and GPR40 RNAi treatment). (F) Effects of GPR40 or GLUT2 RNAi on PPARγ-induced intracellular calcium mobilization and (G) insulin secretion (*n* = 4, ^*^ *P*<0.01 vs. control; ^**^ *P*<0.01 vs. RGZ treatment; **^†^** *P*<0.01 vs. adenoviral PPAR-γ overexpression; and **^††^** *P*<0.01 vs. RGZ treatment+adenoviral PPAR-γ overexpression). (H) 1×10^5^ INS-1 cells were perifused in 3.3 mM glucose for 30 mins at flow rate of 1 ml/min, and then glucose concentrations were modified at 16.7 mM concentration. Fractions were collected at 1 min intervals during 1^st^ peak insulin secretion and then collected at 2 min intervals (*n* = 4, ^*^ *P*<0.05 vs. control; **^†^** *P*<0.01 vs. 10 µM RGZ and GPR40 RNAi treatment).

We first investigated whether or not PPAR-γ activation up-regulated GPR40 expression in INS-1 cells. As shown in Figure 3A, 24 h treatment with RGZ and/or adenoviral PPAR-γ overexpression increased GPR40 expression. In contrast, the level of expression of cyclic-AMP-responsive exchange factor, Epac2, a cAMP sensor for Ca^2+^ mobilization, showed no changes with RGZ and/or PPAR-γ overexpression (Fig. 3A). However, the expression of GPR40 was not changed with 1, 4, and 12 h of treatment with RGZ (Fig. S2A). We next studied whether or not PPAR-γ activation increases calcium mobilization through the GPR40-mediated PLC signaling pathway. As expected, RGZ-mediated intracellular calcium mobilization was significantly reduced with the PLC inhibitor, U-73122, compared to RGZ alone (Fig. 3B), and insulin secretions was significantly decreased with the PLC inhibitors compared to RGZ treatment alone (Fig. 3C).

To further examine the role of GPR40 for RGZ-mediated insulin secretion, we reduced GPR40 expression in INS-1 cells with RNA interference (RNAi) specific for rat GPR40. INS-1 cells expressing RNAi against GPR40 showed nearly 100% reduction in the protein level compared to cells containing control vector, as analyzed by Western blot (Fig. S1B). The RGZ-induced increase in the Ca^2+^ signal was completely abolished in cells transfected with GPR40-specific RNAi. Likewise, insulin secretion was significantly decreased with GPR40 RNAi. Interestingly, U-73122 treatment showed additional inhibitory effects on insulin secretion and intracellular calcium mobilization compared to GPR40 RNAi transfection alone (Fig. 3D, E). Taken together, these results suggest that RGZ increases intracellular calcium mobilization and insulin secretion through the GPR40-mediated PLC signaling pathway in INS-1 cells. In addition, co-treatment with RGZ and PPAR-γ overexpression increased intracellular calcium mobilization and insulin secretion, and was completely blocked by the treatment with GLUT2 RNAi or GPR40 RNAi (Fig. 3F, G).

In perifusion experiments, exposure of the INS-1 cells to 16.7 mM glucose resulted in a rapid increase in 1^st^ and 2^nd^ phase insulin release above basal level. In addition, the addition of 10 µM RGZ potentiated 2.5- and 1.8-fold in 1^st^ and 2^nd^ phase insulin release compared to 16.7 mM glucose alone (*P*<0.05). However, GPR40 RNAi caused a nearly complete inhibition of the insulin release evoked by 10 µM RGZ (Fig. 3H).

### PPAR-γ activation induces the CaMKII and CREB signaling pathways through GPR40 and GLUT2 gene up-regulation

**Figure 4:**
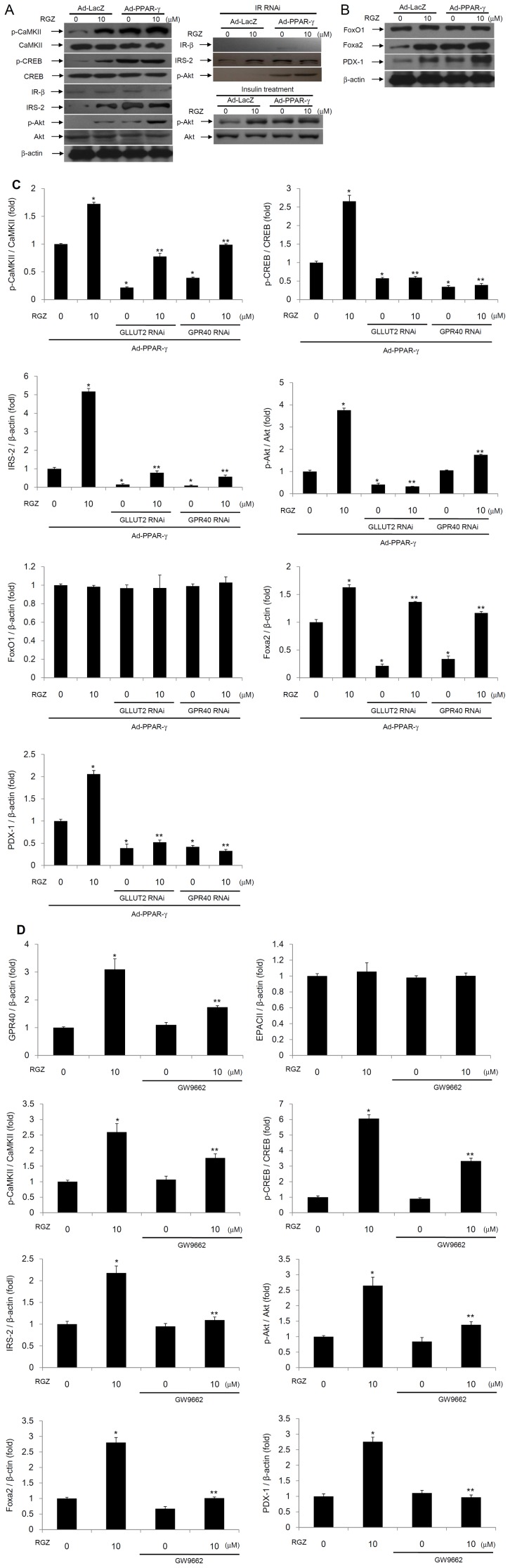
PPAR-γ activation induces the CaMKII and CREB signaling pathways through GPR40 and GLUT2 gene up-regulation. (A, B) Immunoblot for genes involved in CREB signaling pathways and β-cell specific genes with RGZ and/or PPAR-γ overexpression. Immunoblot for IRS-2 and phospho-Akt with insulin receptor-specific RNAi. (C) Effects of GPR40 or GLUT2 RNAi on gene expressions involved in CREB signaling pathways and β-cell function (*n* = 4, ^*^ *P*<0.01 vs. adenoviral PPAR-γ overexpression; ^**^ *P*<0.01 vs. RGZ treatment+adenoviral PPAR-γ overexpression). (D) Effect of co-treatment of 50 µM GW9662, a PPAR-γ antagonist, together with RGZ on the expression levels of GPR40, phospho-CaMKII, phospho-CREB, IRS-2, phospho-Akt, Pdx-1, and FoxA2 (*n* = 4, ^*^ *P*<0.01 vs. control; ^**^ *P*<0.01 vs. RGZ treatment).

Next, we examined the downstream signaling pathway of intracellular calcium mobilization. After treatment with RGZ and/or PPAR-γ overexpression, the phosphorylated levels of CaMKII and downstream CREB were significantly increased, and induced an increase in the levels of IRS-2 and phosphorylated Akt. However, no changes were observed in the level of insulin receptor expression with treatment of RGZ and/or PPAR-γ overexpression (Fig. 4A). As shown above, RGZ and/or PPAR-γ overexpression increased insulin secretion, and thus we examined whether increased levels of IRS-2 and phosphorylated Akt was due to an increased insulin autocrine effect or directly due to the up-regulation of the CaMKII and CREB signaling pathways. After insulin receptor-specific RNAi, expression of insulin receptor was completely blocked, and no increases were noted with RGZ and/or PPAR-γ overexpression in insulin receptor expression. However, the levels of IRS-2 and phosphorylated Akt was increased in the absence of insulin receptor expression with the treatment of RGZ and/or PPAR-γ overexpression (Fig. 4A). Therefore, the increased levels of IRS-2 and phosphorylated Akt by PPAR-γ activation were not by augmented insulin signaling, but by a direct mechanism mediated by up-regulated CaMKII and CREB signaling pathways. In addition, the phosphorylated level of Akt after 10 nM insulin treatment was increased with RGZ and/or PPAR-γ overexpression compared with the non-treated control (Fig. 4A).

We further examined the roles of PPAR-γ activation on β-cell specific gene expression and whether or not it was mediated by GPR40 and GLUT2 up-regulation. After treatment with RGZ and/or PPAR-γ overexpression, the level of expression of the β-cell specific genes, including Pdx-1 and FoxA2, were increased (Fig. 4B), and the GPR40 RNAi or GLUT2 RNAi reduced the levels of phospho-CaMKII, phospho-CREB, IRS-2, phospho-Akt, Pdx-1, and FoxA2 which was mediated by treatment of RGZ and/or PPAR-γ overexpression (Fig. 4C). In addition, the level of expression of BETA2/NeuroD was increased with the treatment of RGZ or adenoviral PPAR-γ overexpression. However, no changes were observed in MafA expression with PPAR-γ activation (data not shown).

To determine whether RGZ treatment up-regulates GPR40 expression in a receptor-dependent manner, we treated 50 µM GW9662 together with RGZ. As a result, the levels of GPR40, phospho-CaMKII, phospho-CREB, IRS-2, phospho-Akt, Pdx-1, and FoxA2 were reduced, and therefore it may be that RGZ treatment increased CaMKII and CREB signaling pathways through GPR40 up-regulation in a receptor-dependent manner (Fig. 4D).

### PPAR-γ activation up-regulates β-cell gene expression through FoxO1 nuclear exclusion

**Figure 5:**
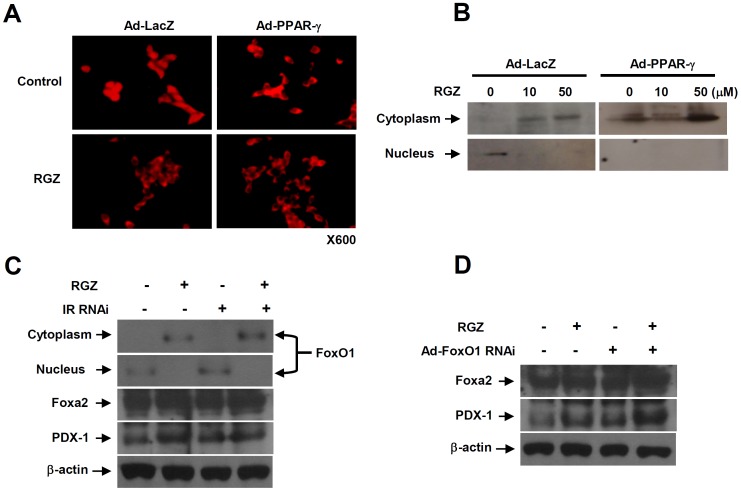
PPAR-γ activation up-regulates β-cell gene expression through FoxO1 nuclear exclusion. (A, B) Immunostaining (A) and immunoblot (B) of nuclear-cytoplasm shuttling for FoxO1 with RGZ treatment and/or PPAR-γ overexpression. (C) Immunoblot for β-cell specific gene expressions and FoxO1 shuttling with insulin receptor RNAi. (D) Immunoblot for β-cell specific gene expressions after treatment with adenovirus containing rat FoxO1 shRNA.

We examined whether PPAR-γ activation affects the nuclear-cytoplasm shuttling of FoxO1. Under basal condition (serum-free 16.7 mM glucose in RPMI-1640 media), FoxO1 was exclusively located in the nucleus; however, PPAR-γ activation with RGZ and/or PPAR-γ overexpression translocated FoxO1 from nucleus to cytoplasm in INS-1 cells (Fig. 5A). In agreement with immunofluorescence staining results, FoxO1 was translocated from the nucleus to the cytoplasm, and exclusively located in the cytoplasm with RGZ and/or PPAR-γ overexpression in Western blotting (Fig. 5B).

Next, we examined whether or not PPAR-γ-mediated FoxO1 nuclear exclusion is dependent on PPAR-γ-mediated insulin secretion. Even though the expression of insulin receptor was completely blocked with insulin receptor RNAi, FoxO1 was translocated to the cytoplasm with RGZ treatment (Fig. 5C). In addition, the expression of β-cell genes, including FoxA2 and Pdx-1, was up-regulated with RGZ treatment, despite the insulin receptor RNAi, and combined treatment with RGZ and FoxO1 RNAi showed a synergetic effect on β-cell gene expression compared to either RGZ treatment or FoxO1 RNAi alone (Fig. 5C, D).

### RGZ treatment prevents lipotoxic and ER stress-induced β-cell apoptosis

**Figure 6:**
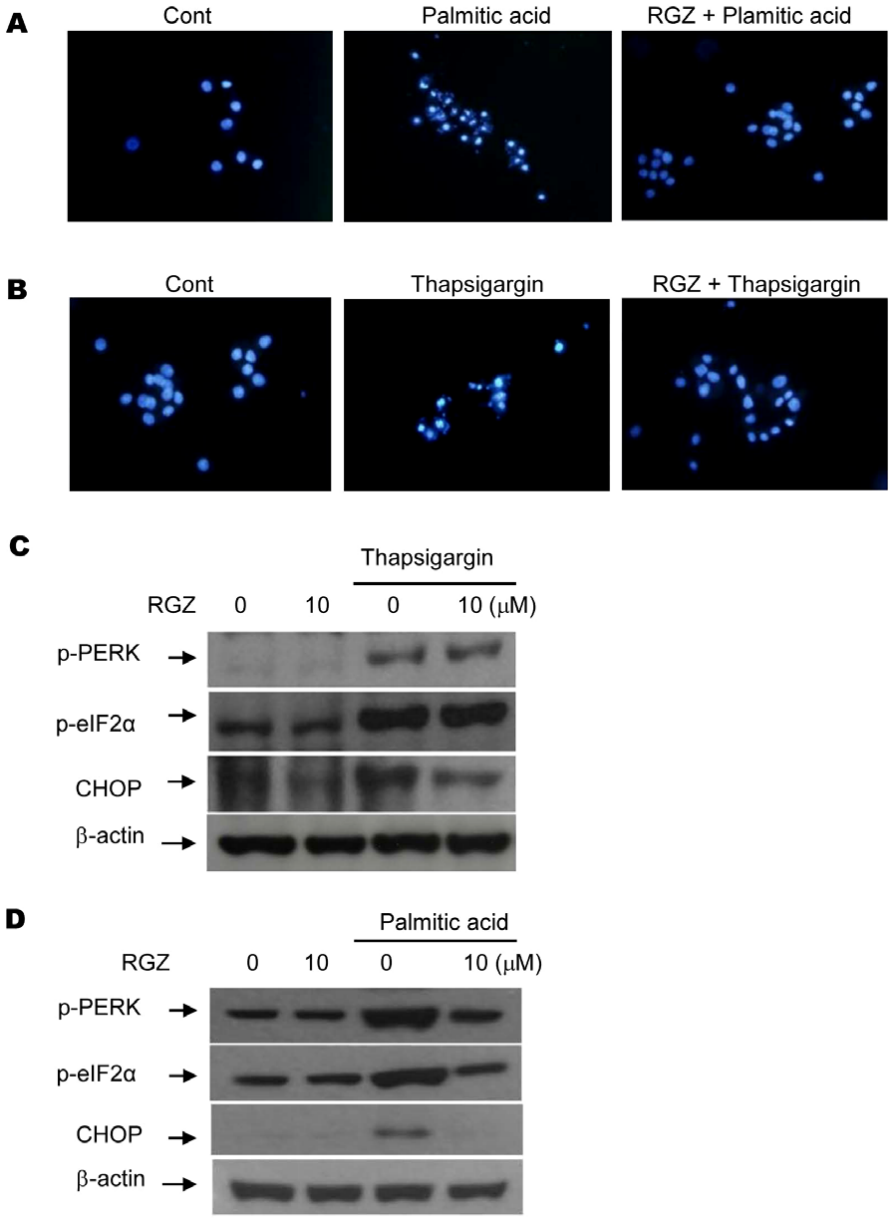
RGZ treatment prevents lipotoxic and ER stress-induced β-cell apoptosis. (A, B) INS-1 cells were pretreated with or without RGZ (10 µM) and challenged with palmitate (1.0 mM) or thapsigargin (50 µM) for 24 h. Cell apoptosis was examined by Hoechst staining. (C) Effect of RGZ treatment on CHOP expression induced by thapsigargin (50 µM). (D) Effect of RGZ treatment on CHOP expression induced by palmitate (1.0 mM).

To determine the role of RGZ on β-cell death, we treated INS-1 cells with 1.0 mM palmitate or 50 µM thapsigargin for 24 h and assessed β-cell apoptosis with Hoechst 33258 staining. The number of fluorescence-stained cells increased with lipotoxic or ER stress conditions compared with control cells; however, the addition of RGZ with palmitate or thapsigargin treatment reduced β-cell apoptosis (Fig. 6A, B). In support of β-cell apoptosis prevention, RGZ treatment reduced the level of expression of CHOP induced by thapsigargin treatment. In addition, RGZ treatment reduced the levels of expression of ER stress markers including p-PERK, p-eIF2α, and CHOP induced by 1.0 mM palmitate (Fig. 6C, D).

### PPAR-γ activation increases GPR40 expression in primary rat islets and OLETF rats

**Figure 7:**
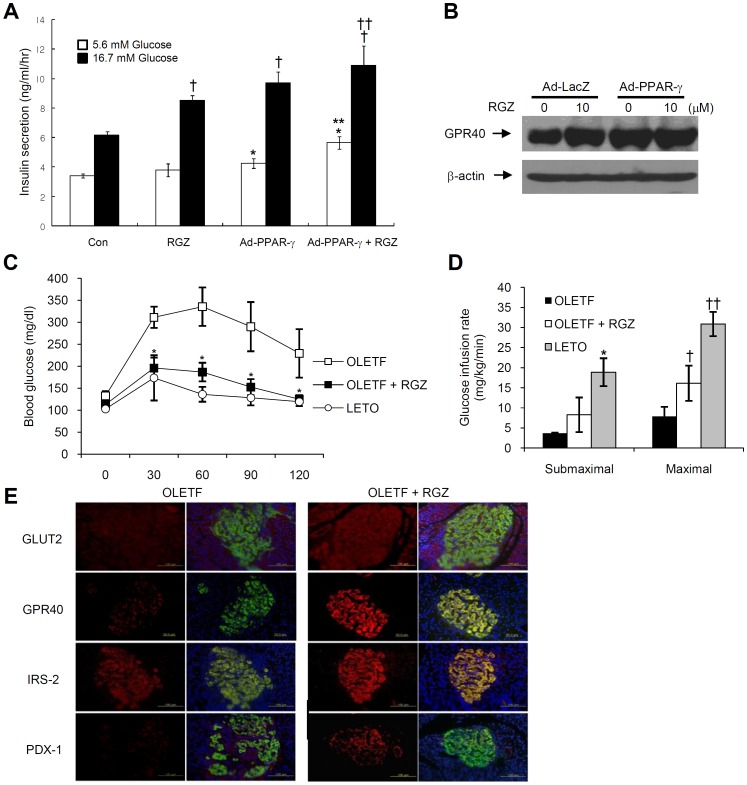
PPAR-γ activation increases GPR40 expression in primary rat islets and OLETF rats. (A) Primary rat islets were treated with 10 µM RGZ for 24 h, and incubated for an additional 1 h in 5.6 or 16.7 mM of glucose. Insulin was determined using a rat insulin ELISA kit (ANOVA within same glucose conditions: *n* = 4, ^*^ *P*<0.05 vs. control with 5.6 mM glucose; ^**^ *P*<0.01 vs. RGZ or adenoviral PPAR-γ overexpression with 5.6 mM glucose; **^†^** *P*<0.01 vs. control with 16.7 mM glucose; and **^††^** *P*<0.01 vs. RGZ with 16.7 mM glucose). (B) Immunoblot for GPR40 expression with 10 µM RGZ treatment and/or adenoviral PPAR-γ overexpression. (C) At 10 weeks of age, OLETF rats were randomly assigned to the RGZ treatment or control group, and 3 mg/kg of RGZ was given by mouth through gavage. After 14 weeks of RGZ treatment (24 weeks of age), oral glucose tolerance test was performed (*n* = 4, ^*^ *P*<0.05 vs. OLETF rats). (D) At 24 weeks of age, hyperinsulinemic euglycemic clamping was performed and glucose infusion rate was measured. (ANOVA within same groups: *n* = 4, ^*^ *P*<0.05 vs. OLETF rats or OLETF rats+RGZ; ^**^ *P*<0.05 vs. OLETF rats). (E) Immunohistochemical staining of GLUT2, IRS-2, Pdx-1, and GPR40.

In agreement with the results of INS-1 cells, 24 h treatment with 10 µM RGZ and/or adenoviral PPAR-γ overexpression increased GPR40 expression in primary islets. In addition, PPAR-γ activation with RGZ and/or adenoviral PPAR-γ overexpression increased GSIS in high (16.7 mM) glucose conditions. However, GSIS did not increase in RGZ treatment in the case of basal (5.6 mM) glucose conditions (Fig. 7A, B). We next performed metabolic tests including OGTT and euglycemic clamping. RGZ treated OLEFT rats showed significantly reduced blood glucose levels compared with non-treated OLEFT rats (Fig. 7C). To assess the insulin sensitivity in OLETF rats, hyperinsulinemic-euglycemic clamp studies were performed. Maximal glucose infusion rate (GIR) was significantly higher in RGZ-treated OLETF rats (n = 4) compared with untreated OLETF rats (n = 4) at 24 weeks (7.91±2.32 mg/kg/min in OLETF rats and 16.10±4.39 mg/kg/min in RGZ-treated OLETF rats, *P*<0.05). However, there was no significant difference between treated and untreated OLETF rats with submaximal GIR (Fig. 7D). In immunohistochemical staining, RGZ-treated islets were relatively well-preserved, and there was stronger positive insulin staining. In addition, GLUT2, IRS-2, Pdx-1, and GPR40 were more strongly expressed in RGZ-treated islets compared to control islets (Fig. 7E).

## Discussion

There is mounting evidence that TZDs protect pancreatic β-cells from a variety of noxious stimuli, including excessive nutrients, cytokines, h-IAPP, and ER stress [11]–[19], and a recent human study also concurs with the protective role of TZD on β-cell function compared to other anti-diabetic drugs, such as sulfonylurea and metformin [34]. However, it is still obscure as to the precise mechanisms of PPAR-γ activation that regulate β-cell function and mass, and although there are many studies that TZDs protect β-cells from lipoapoptosis in diabetic rats [17], it is still elusive whether the protective role of TZDs is direct or secondary to lowered blood glucose from improved insulin sensitivity. Moreover, there is significant controversy about whether or not PPAR-γ activation increases GSIS in pancreatic β-cells. Some of the previous studies have shown that PPAR-γ activation with TZDs and/or PPAR-γ overexpression is detrimental to β-cell function, and suppresses insulin secretion and proinsulin biosynthesis [7], [35]–[38]. For example, it has been reported that troglitazone activates AMP-activated protein kinase and inhibits insulin secretion from MIN6 cells [37], and PPAR-γ overexpression suppresses insulin secretion in isolated pancreatic islets through induction of UCP-2 protein [35]. Moreover, Schinner *et al*. [39] reported that human insulin gene promoter activity is inhibited by RGZ and PPAR-γ. However, other studies have demonstrated the contradictory results of TZDs or PPAR-γ, which showed the PPAR-γ-stimulated insulin secretion in pancreatic β-cells [3], [8]–[10]. In terms of β-cell gene expression, Moibi *et al*. [6] showed that Pdx-1, Nkx6.1, glucokinase and GLUT2 were increased after treatment with troglitazone for 3 days in INS-1 cells, and knockdown of PPAR-γ with RNAi lowered the mRNA levels of Pdx-1, glucokinase, GLUT2, and proinsulin II by more than half. Moreover, it was reported that some of the key β-cell genes, including Pdx-1, GLUT2, and glucokinase, have PPRE in the promoter region [4], [5], [40].

In the current study, we showed that RGZ and/or adenoviral PPAR-γ overexpression increase insulin secretion in high glucose conditions, and intracellular calcium mobilization is important in this step. Therefore, treatment with nifedipine and thapsigargin that blocked the extracellular and intracellular calcium sources, respectively, decreased RGZ-induced insulin secretion. Apart from insulin secretion, RGZ and/or PPAR-γ overexpression increase β-cell gene expression including Pdx-1, FoxA2, and BETA2/NeuroD. Similar to our results, Richardson *et al*. [41] showed that >24 h treatment with RGZ promoted the nuclear accumulation of IPF1 and FoxA2 independent of glucose concentration, and stimulated a two-fold increase in the activity of the Ipf1 gene promoter. Several explanations are possible regarding the mechanism of PPAR-γ-induced β-cell gene expression. First, putative PPRE was identified in the mouse Pdx-1 promoter, and TZDs increase Pdx-1 expression through a PPAR-γ-mediated mechanism [4]. Second, it may be possible that the increased Pdx-1 and FoxA2 expression is due to FoxO1 nuclear exclusion by PPAR-γ activation. Because FoxO1 and FoxA2 share common DNA binding sites in the Pdx-1 promoter, they compete with each other for binding to the Pdx-1 promoter [42]. Therefore, RGZ-induced FoxO1 nuclear exclusion leads to increase FoxA2 binding to the Pdx-1 promoter, resulting up-regulation of Pdx-1 gene expression. To exclude the possibility that RGZ treatment induces FoxO1 nuclear exclusion merely through the increased insulin secretion, we blocked the insulin signaling pathway via insulin receptor RNAi, and assessed FoxO1 cellular localization. Although, the expression of insulin receptor was completely blocked, FoxO1 was translocated from the nucleus to the cytoplasm with the RGZ treatment, and thus it seems that there may be a direct mechanism in PPAR-γ-induced FoxO1 nuclear exclusion rather than PI3K–Akt pathway mediated FoxO1 shuttling. In agreement with our findings, Dowell *et al*. [43] reported that FoxO1 was identified as a PPAR-γ-interacting protein in a yeast two-hybrid screen, and PPAR-γ and RXRα expression vector and/or RGZ treatment resulted in a dose-dependent inhibition of Foxo1-driven reporter activity. Interestingly, PPARγ-induced increased calcium mobilization and insulin secretion is mediated by FFA receptor GPR40 gene induction, and was completely blocked by the PLC inhibitor, U-73122, or direct GPR40 RNAi. This is the first evidence showing the novel mechanism of PPAR-γ that increases insulin secretion in pancreatic β-cells. In addition, the expression of Pdx-1 and FoxA2 following RGZ treatment and/or PPAR-γ overexpression was up-regulated in a GPR40-dependent manner. In agreement with our findings, it has been reported that the HR region in the 5′-flanking region of the GPR40 gene showed a strong β-cell-specific enhancer activity, and can bind the Pdx-1 and BETA2 both *in vitro* and *in vivo* [44].

Recently, Gras *et al*. [32] reported that TZDs bind to the GPR40 receptor, resulting in Ca^2+^ mobilization from ER calcium stores, and which induce the proliferation of non-malignant human bronchial epithelial cells. However, the non-TZD PPAR-γ ligand, 15 d-PGJ_2_, did not induce any Ca^2+^ signal and instead inhibited cell growth. Collectively, this study suggested that TZDs increase intracellular calcium through the GPR40 activation in a receptor-independent manner. The receptor-independent effects of TZDs include anti-inflammatory actions, anti-proliferative effects and cell apoptosis, inhibition of mitochondrial function, and modification of energy metabolism, and based on several findings include the following: (1) the concentrations needed to demonstrate TZD actions were much greater than the reported EC50 values; (2) non-TZD agonists showed little or no effect; (3) PPAR-γ antagonists did not block TZD effects; (4) effects occurred rapidly (within minutes to hours); and (5) effects occurred in the absence of PPAR-γ expression or of PPRE in gene promoters [45]. However, GPR40 induction through PPAR-γ activation was shown to be a receptor-dependent effect in ours. First, we demonstrated increased GPR40 expression and subsequent insulin secretion with RGZ treatment for 24 h, and no changes in GPR40 expression and insulin secretion were noted with short-term (1, 4, and 12 h) treatment (Fig. S2A, B). Second, GW9662, a PPAR-γ antagonist, completely blocked RGZ-induced GSIS and insulin biosynthesis in our previous report [9], and the levels of GPR40, phospho-CaMKII, phospho-CREB, IRS-2, phospho-Akt, Pdx-1, and FoxA2 were reduced by GW9662, and therefore it is more likely that PPAR-γ activation induces GPR40 expression through receptor-dependent manner in pancreatic β-cells. In this study, although short-term (less than 24 h) treatment with RGZ did not increase GPR40 expression and insulin secretion in pancreatic β-cells, 10 µM of PGJ_2_ for 10 minutes increased intracellular calcium mobilization and insulin secretion. In contrast, 24 h treatment with PGJ_2_ did not show any intracellular calcium mobilization or GPR40, phospho-CaMKII, phospho-CREB, IRS-2, phospho-Akt, Pdx-1, and FoxA2 induction (data not shown). In contrast to our results, 1 h exposure to FFAs significantly enhanced GSIS and increased expression of PDX-1 and GLUT2 in pSilencer-control transfected cells, but not in cells transfected with GPR40shRNA. While long term (48 h) exposure to FFAs significantly impaired GSIS in control and GPR40shRNA cells. Furthermore, pioglitazone enhanced insulin secretion in pSilencer-control transfected cells exposed to FFAs for 48 h, but not in cells transfected with GPR40shRNA [46]. Although we do not know why there is a discrepancy in the mechanism of GPR40 induction through PPAR-γ activation, it may be primarily due to the difference in cell type and experimental conditions.

Despite the link between GPR40 and PPAR-γ mediated insulin secretion, another mechanism still can be considered. For example, it has recently been reported that the ATP-binding cassette transporter A1 (ABCA1), a cellular cholesterol transporter, in beta cell cholesterol homeostasis and insulin secretion. Briefly, mice with specific inactivation of Abca1 in beta cells showed marked impairment of glucose tolerance and defective insulin secretion. Importantly, RGZ up-regulated Abca1 in beta-cells, and reduced islet free cholesterol levels in wild-type mice, but not beta-cell specific Abca1 lacking mice. This was associated with significantly improved glucose tolerance in wild-type mice with no effect of RGZ on glucose tolerance in mice deficient for beta-cell Abca1. Therefore, in addition to the up-regulation of GPR40, PPAR-gamma may activate beta-cell Abca1 and subsequent reduction of islet fat content is an important mechanism by which RGZ increases insulin secretion [47], [48].

In the present study, the phosphorylated levels of CaMKII and CREB, IRS-2, and phospho-Akt were up-regulated by PPAR-γ activation. To determine whether or not the increased IRS-2 and phospho-Akt was due to increased insulin signaling or directly due to the up-regulation of the CaMKII and CREB signaling pathway, we knocked down the insulin receptor with RNAi. Even after insulin receptor RNAi, IRS-2 and phospho-Akt levels were increased with treatment of RGZ and/or PPAR-γ overexpression. Therefore, increased IRS-2 and phospho-Akt levels are direct through the up-regulated CaMKII and CREB signaling pathways and these results may partly explain the protective role of RGZ from lipotoxic or ER stress induced β-cell apoptosis in this study.

In contrast to the short-term effect of FFAs on insulin secretion, long-term exposure of islets to FFAs results in impaired GSIS through intracellular accumulation of lipid signaling molecules that inhibit insulin secretion [23]. Therefore, some studies questioned the beneficial role of GPR40 on β-cell function. Lan *et al*. [49] suggested that even though GPR40 is required for normal insulin secretion in response to FFAs, *Ffar1^+/+^* and *Ffar1^−/−^* mice had similar weight, adiposity, hyperinsulinemia, and lipid accumulation in livers on high-fat diets. Moreover, Steneberg *et al*. [50] showed that GPR40-deficient β-cells secrete less insulin in response to FFAs, and loss of GPR40 protects mice from obesity-induced metabolic derangements. Conversely, overexpression of GPR40 in β-cells leads to impaired β-cell function, hypoinsulinemia, and diabetes, therefore, the authors suggested that GPR40 may play a key role in the development of type 2 diabetes. Furthermore, it was reported that GPR40 activation may have the potential to inhibit insulin secretion through the opening of ATP-dependent K^+^ channels in rat β-cells [51]. At present, we could not explain the reason why the studies showed a different role of GPR40 from our results, and therefore further studies are necessary to clarify the role of GPR40 on pancreatic β-cell function following stimulation with FFAs and during the development of diabetes.

In conclusion, our results demonstrate that PPAR-γ activation through TZDs and/or adenoviral overexpression increases intracellular calcium mobilization and insulin secretion in INS-1 cells, and which was mediated by the up-regulation of FFA receptor GPR40 and GLUT2 expression. Moreover, the PPAR-γ-mediated GPR40 activation increased the level of expression of β-cell genes, including Pdx-1 and FoxA2.

## Supporting Information

Figure S1 (A) Overexpression of PPAR- γ and suppression of FoxO1 in INS-1 cells. Efficiency of adenovirus was determined by X-gal staining, GFP expression, and Western blotting. (B) Inhibition of GPR40, GLUT2, and IR-β expression with RNAi transfection. As the RNAi concentrations increased, target protein expressions were suppressed with RNAi transfection.
(TIF)

Figure S2 (A) Acute effect of rosiglitazone on GPR40 expression in INS-1 cells. (B) Acute effect of rosiglitazone on insulin secretion in INS-1 cells (*n* = 4, ^*^ *P*<0.01 vs. 0 hr).
(TIF)

## References

[1] Ferre P (2004) The biology of peroxisome proliferator-activated receptors: relationship with lipid metabolism and insulin sensitivity. Diabetes 53 Suppl 1: S43–S50. 14749265 10.2337/diabetes.53.2007.s43

[2] Semple RK, Chatterjee VK, O'Rahilly S (2006) PPAR gamma and human metabolic disease. J Clin Invest 116: 581–589. 16511590 10.1172/JCI28003 PMC1386124

[3] Santini E, Fallahi P, Ferrari SM, Masoni A, Antonelli A, et al (2004) Effect of PPAR-gamma activation and inhibition on glucose-stimulated insulin release in INS-1e cells. Diabetes 53 Suppl 3: S79–S83. 15561927 10.2337/diabetes.53.suppl_3.s79

[4] Gupta D, Jetton TL, Mortensen RM, Duan SZ, Peshavaria M, et al (2008) In vivo and in vitro studies of a functional peroxisome proliferator-activated receptor gamma response element in the mouse pdx-1 promoter. J Biol Chem 283: 32462–32470. 18718916 10.1074/jbc.M801813200 PMC2583321

[5] Kim HI, Cha JY, Kim SY, Kim JW, Roh KJ, et al (2002) Peroxisomal proliferator-activated receptor-gamma upregulates glucokinase gene expression in beta-cells. Diabetes 51: 676–685. 11872666 10.2337/diabetes.51.3.676

[6] Moibi JA, Gupta D, Jetton TL, Peshavaria M, Desai R, et al (2007) Peroxisome proliferator-activated receptor-gamma regulates expression of PDX-1 and NKX6.1 in INS-1 cells. Diabetes 56: 88–95. 17192469 10.2337/db06-0948

[7] Nakamichi Y, Kikuta T, Ito E, Ohara-Imaizumi M, Nishiwaki C, et al (2003) PPAR-gamma overexpression suppresses glucose-induced proinsulin biosynthesis and insulin release synergistically with pioglitazone in MIN6 cells. Biochem Biophys Res Commun 306: 832–836. 12821117 10.1016/s0006-291x(03)01045-3

[8] Yang C, Chang TJ, Chang JC, Liu MW, Tai TY, et al (2001) Rosiglitazone (BRL 49653) enhances insulin secretory response via phosphatidylinositol 3-kinase pathway. Diabetes 50: 2598–2602. 11679440 10.2337/diabetes.50.11.2598

[9] Kim HS, Noh JH, Hong SH, Hwang YC, Yang TY, et al (2008) Rosiglitazone stimulates the release and synthesis of insulin by enhancing GLUT-2, glucokinase and BETA2/NeuroD expression. Biochem Biophys Res Commun 367: 623–629. 18191635 10.1016/j.bbrc.2007.12.192

[10] Chang TJ, Chen WP, Yang C, Lu PH, Liang YC, et al (2009) Serine-385 phosphorylation of inwardly rectifying K+ channel subunit (Kir6.2) by AMP-dependent protein kinase plays a key role in rosiglitazone-induced closure of the K(ATP) channel and insulin secretion in rats. Diabetologia 52: 1112–1121. 19357830 10.1007/s00125-009-1337-4

[11] Kim EK, Kwon KB, Koo BS, Han MJ, Song MY, et al (2007) Activation of peroxisome proliferator-activated receptor-gamma protects pancreatic beta-cells from cytokine-induced cytotoxicity via NF kappaB pathway. Int J Biochem Cell Biol 39: 1260–1275. 17521952 10.1016/j.biocel.2007.04.005

[12] Maedler K, Sergeev P, Ris F, Oberholzer J, Joller-Jemelka HI, et al (2002) Glucose-induced beta cell production of IL-1beta contributes to glucotoxicity in human pancreatic islets. J Clin Invest 110: 851–860. 12235117 10.1172/JCI15318 PMC151125

[13] Lin CY, Gurlo T, Haataja L, Hsueh WA, Butler PC (2005) Activation of peroxisome proliferator-activated receptor-gamma by rosiglitazone protects human islet cells against human islet amyloid polypeptide toxicity by a phosphatidylinositol 3′-kinase-dependent pathway. J Clin Endocrinol Metab 90: 6678–6686. 16204373 10.1210/jc.2005-0079

[14] Hull RL, Shen ZP, Watts MR, Kodama K, Carr DB, et al (2005) Long-term treatment with rosiglitazone and metformin reduces the extent of, but does not prevent, islet amyloid deposition in mice expressing the gene for human islet amyloid polypeptide. Diabetes 54: 2235–2244. 15983227 10.2337/diabetes.54.7.2235

[15] Matsui J, Terauchi Y, Kubota N, Takamoto I, Eto K, et al (2004) Pioglitazone reduces islet triglyceride content and restores impaired glucose-stimulated insulin secretion in heterozygous peroxisome proliferator-activated receptor-gamma-deficient mice on a high-fat diet. Diabetes 53: 2844–2854. 15504964 10.2337/diabetes.53.11.2844

[16] Unger RH, Zhou YT (2001) Lipotoxicity of beta-cells in obesity and in other causes of fatty acid spillover. Diabetes 50 Suppl 1: S118–121. 11272168 10.2337/diabetes.50.2007.s118

[17] Shimabukuro M, Zhou YT, Lee Y, Unger RH (1998) Troglitazone lowers islet fat and restores beta cell function of Zucker diabetic fatty rats. J Biol Chem 273: 3547–3550. 9452481 10.1074/jbc.273.6.3547

[18] Higa M, Zhou YT, Ravazzola M, Baetens D, Orci L, et al (1999) Troglitazone prevents mitochondrial alterations, beta cell destruction, and diabetes in obese prediabetic rats. Proc Natl Acad Sci U S A 96: 11513–11518. 10500208 10.1073/pnas.96.20.11513 PMC18065

[19] Evans-Molina C, Robbins RD, Kono T, Tersey SA, Vestermark GL, et al (2009) Peroxisome proliferator-activated receptor gamma activation restores islet function in diabetic mice through reduction of endoplasmic reticulum stress and maintenance of euchromatin structure. Mol Cell Biol 29: 2053–2067. 19237535 10.1128/MCB.01179-08 PMC2663298

[20] Briscoe CP, Tadayyon M, Andrews JL, Benson WG, Chambers JK, et al (2003) The orphan G protein-coupled receptor GPR40 is activated by medium and long chain fatty acids. J Biol Chem 278: 11303–11311. 12496284 10.1074/jbc.M211495200

[21] Itoh Y, Kawamata Y, Harada M, Kobayashi M, Fujii R, et al (2003) Free fatty acids regulate insulin secretion from pancreatic beta cells through GPR40. Nature 422: 173–176. 12629551 10.1038/nature01478

[22] Kotarsky K, Nilsson NE, Flodgren E, Owman C, Olde B (2003) A human cell surface receptor activated by free fatty acids and thiazolidinedione drugs. Biochem Biophys Res Commun 301: 406–410. 12565875 10.1016/s0006-291x(02)03064-4

[23] Tomita T, Masuzaki H, Iwakura H, Fujikura J, Noguchi M, et al (2006) Expression of the gene for a membrane-bound fatty acid receptor in the pancreas and islet cell tumours in humans: evidence for GPR40 expression in pancreatic beta cells and implications for insulin secretion. Diabetologia 49: 962–968. 16525841 10.1007/s00125-006-0193-8

[24] Feng DD, Luo Z, Roh SG, Hernandez M, Tawadros N, et al (2006) Reduction in voltage-gated K+ currents in primary cultured rat pancreatic beta-cells by linoleic acids. Endocrinology 147: 674–682. 16254037 10.1210/en.2005-0225

[25] Fujiwara K, Maekawa F, Yada T (2005) Oleic acid interacts with GPR40 to induce Ca2+ signaling in rat islet beta-cells: mediation by PLC and L-type Ca2+ channel and link to insulin release. Am J Physiol Endocrinol Metab 289: E670–677. 15914509 10.1152/ajpendo.00035.2005

[26] Schnell S, Schaefer M, Schofl C (2007) Free fatty acids increase cytosolic free calcium and stimulate insulin secretion from beta-cells through activation of GPR40. Mol Cell Endocrinol 263: 173–180. 17101212 10.1016/j.mce.2006.09.013

[27] Latour MG, Alquier T, Oseid E, Tremblay C, Jetton TL, et al (2007) GPR40 is necessary but not sufficient for fatty acid stimulation of insulin secretion in vivo. Diabetes 56: 1087–1094. 17395749 10.2337/db06-1532 PMC1853382

[28] Vettor R, Granzotto M, De Stefani D, Trevellin E, Rossato M, et al (2008) Loss-of-function mutation of the GPR40 gene associates with abnormal stimulated insulin secretion by acting on intracellular calcium mobilization. J Clin Endocrinol Metab 93: 3541–3550. 18583466 10.1210/jc.2007-2680

[29] Nagasumi K, Esaki R, Iwachidow K, Yasuhara Y, Ogi K, et al (2009) Overexpression of GPR40 in pancreatic beta-cells augments glucose-stimulated insulin secretion and improves glucose tolerance in normal and diabetic mice. Diabetes 58: 1067–1076. 19401434 10.2337/db08-1233 PMC2671040

[30] Gromada J (2006) The free fatty acid receptor GPR40 generates excitement in pancreatic beta-cells. Endocrinology 147: 672–673. 16418431 10.1210/en.2005-1388

[31] Edfalk S, Steneberg P, Edlund H (2008) Gpr40 is expressed in enteroendocrine cells and mediates free fatty acid stimulation of incretin secretion. Diabetes 57: 2280–2287. 18519800 10.2337/db08-0307 PMC2518478

[32] Gras D, Chanez P, Urbach V, Vachier I, Godard P, et al (2009) Thiazolidinediones induce proliferation of human bronchial epithelial cells through the GPR40 receptor. Am J Physiol Lung Cell Mol Physiol 296: L970–978. 19346435 10.1152/ajplung.90219.2008

[33] Asfari M, Janjic D, Meda P, Li G, Halban PA, et al (1992) Establishment of 2-mercaptoethanol-dependent differentiated insulin-secreting cell lines. Endocrinology 130: 167–178. 1370150 10.1210/endo.130.1.1370150

[34] Kahn SE, Haffner SM, Heise MA, Herman WH, Holman RR, et al (2006) Glycemic durability of rosiglitazone, metformin, or glyburide monotherapy. N Engl J Med 355: 2427–2443. 17145742 10.1056/NEJMoa066224

[35] Ito E, Ozawa S, Takahashi K, Tanaka T, Katsuta H, et al (2004) PPAR-gamma overexpression selectively suppresses insulin secretory capacity in isolated pancreatic islets through induction of UCP-2 protein. Biochem Biophys Res Commun 324: 810–814. 15474499 10.1016/j.bbrc.2004.08.238

[36] Ravnskjaer K, Boergesen M, Rubi B, Larsen JK, Nielsen T, et al (2005) Peroxisome proliferator-activated receptor alpha (PPARalpha) potentiates, whereas PPARgamma attenuates, glucose-stimulated insulin secretion in pancreatic beta-cells. Endocrinology 146: 3266–3276. 15878969 10.1210/en.2004-1430

[37] Wang X, Zhou L, Shao L, Qian L, Fu X, et al (2007) Troglitazone acutely activates AMP-activated protein kinase and inhibits insulin secretion from beta cells. Life Sci 81: 160–165. 17544010 10.1016/j.lfs.2007.04.034

[38] Bollheimer LC, Troll S, Landauer H, Wrede CE, Schölmerich J, et al (2003) Insulin-sparing effects of troglitazone in rat pancreatic islets. J Mol Endocrinol 31: 61–69. 12914525 10.1677/jme.0.0310061

[39] Schinner S, Krätzner R, Baun D, Dickel C, Blume R, et al (2009) Inhibition of human insulin gene transcription by peroxisome proliferator-activated receptor gamma and thiazolidinedione oral antidiabetic drugs. Br J Pharmacol 157: 736–745. 19338578 10.1111/j.1476-5381.2009.00208.x PMC2721259

[40] Kim HI, Kim JW, Kim SH, Cha JY, Kim KS, et al (2000) Identification and functional characterization of the peroxisomal proliferator response element in rat GLUT2 promoter. Diabetes 49: 1517–1524. 10969836 10.2337/diabetes.49.9.1517

[41] Richardson H, Campbell SC, Smith SA, Macfarlane WM (2006) Effects of rosiglitazone and metformin on pancreatic beta cell gene expression. Diabetologia 49: 685–696. 16489446 10.1007/s00125-006-0155-1

[42] Kitamura T, Nakae J, Kitamura Y, Kido Y, Biggs WH 3rd, et al (2002) The forkhead transcription factor Foxo1 links insulin signaling to Pdx1 regulation of pancreatic beta cell growth. J Clin Invest 110: 1839–1847. 12488434 10.1172/JCI200216857 PMC151657

[43] Dowell P, Otto TC, Adi S, Lane MD (2003) Convergence of peroxisome proliferator-activated receptor gamma and Foxo1 signaling pathways. J Biol Chem 278: 45485–45491. 12966085 10.1074/jbc.M309069200

[44] Bartoov-Shifman R, Ridner G, Bahar K, Rubins N, Walker MD (2007) Regulation of the gene encoding GPR40, a fatty acid receptor expressed selectively in pancreatic beta cells. J Biol Chem 282: 23561–23571. 17525159 10.1074/jbc.M702115200

[45] Feinstein DL, Spagnolo A, Akar C, Weinberg G, Murphy P, et al (2005) Receptor-independent actions of PPAR thiazolidinedione agonists: is mitochondrial function the key? Biochem Pharmacol 70: 177–188. 15925327 10.1016/j.bcp.2005.03.033

[46] Wu P, Yang L, Shen X (2010) The relationship between GPR40 and lipotoxicity of the pancreatic β-cells as well as the effect of pioglitazone. Biochem Biophys Res Commun 403: 36–39. 21036144 10.1016/j.bbrc.2010.10.105

[47] Brunham LR, Kruit JK, Pape TD, Timmins JM, Reuwer AQ, et al (2007) Beta-cell ABCA1 influences insulin secretion, glucose homeostasis and response to thiazolidinedione treatment. Nat Med 13: 340–347. 17322896 10.1038/nm1546

[48] Kruit JK, Kremer PH, Dai L, Tang R, Ruddle P, et al (2010) Cholesterol efflux via ATP-binding cassette transporter A1 (ABCA1) and cholesterol uptake via the LDL receptor influences cholesterol-induced impairment of beta cell function in mice. Diabetologia 53: 1110–1119. 20229095 10.1007/s00125-010-1691-2

[49] Lan H, Hoos LM, Liu L, Tetzloff G, Hu W, et al (2008) Lack of FFAR1/GPR40 does not protect mice from high-fat diet-induced metabolic disease. Diabetes 57: 2999–3006. 18678612 10.2337/db08-0596 PMC2570396

[50] Steneberg P, Rubins N, Bartoov-Shifman R, Walker MD, Edlund H (2005) The FFA receptor GPR40 links hyperinsulinemia, hepatic steatosis, and impaired glucose homeostasis in mouse. Cell Metab 1: 245–258. 16054069 10.1016/j.cmet.2005.03.007

[51] Zhao YF, Pei J, Chen C (2008) Activation of ATP-sensitive potassium channels in rat pancreatic beta-cells by linoleic acid through both intracellular metabolites and membrane receptor signalling pathway. J Endocrinol 198: 533–540. 18550787 10.1677/JOE-08-0105

