# Supplementary figures and images for: PPAR-γ Activation Increases Insulin Secretion through the Up-regulation of the Free Fatty Acid Receptor GPR40 in Pancreatic β-Cells
Source: PLoS One. 2013 Jan 23;8(1):e50128. doi: 10.1371/journal.pone.0050128 (PMC3553172; doi:10.1371/journal.pone.0050128)

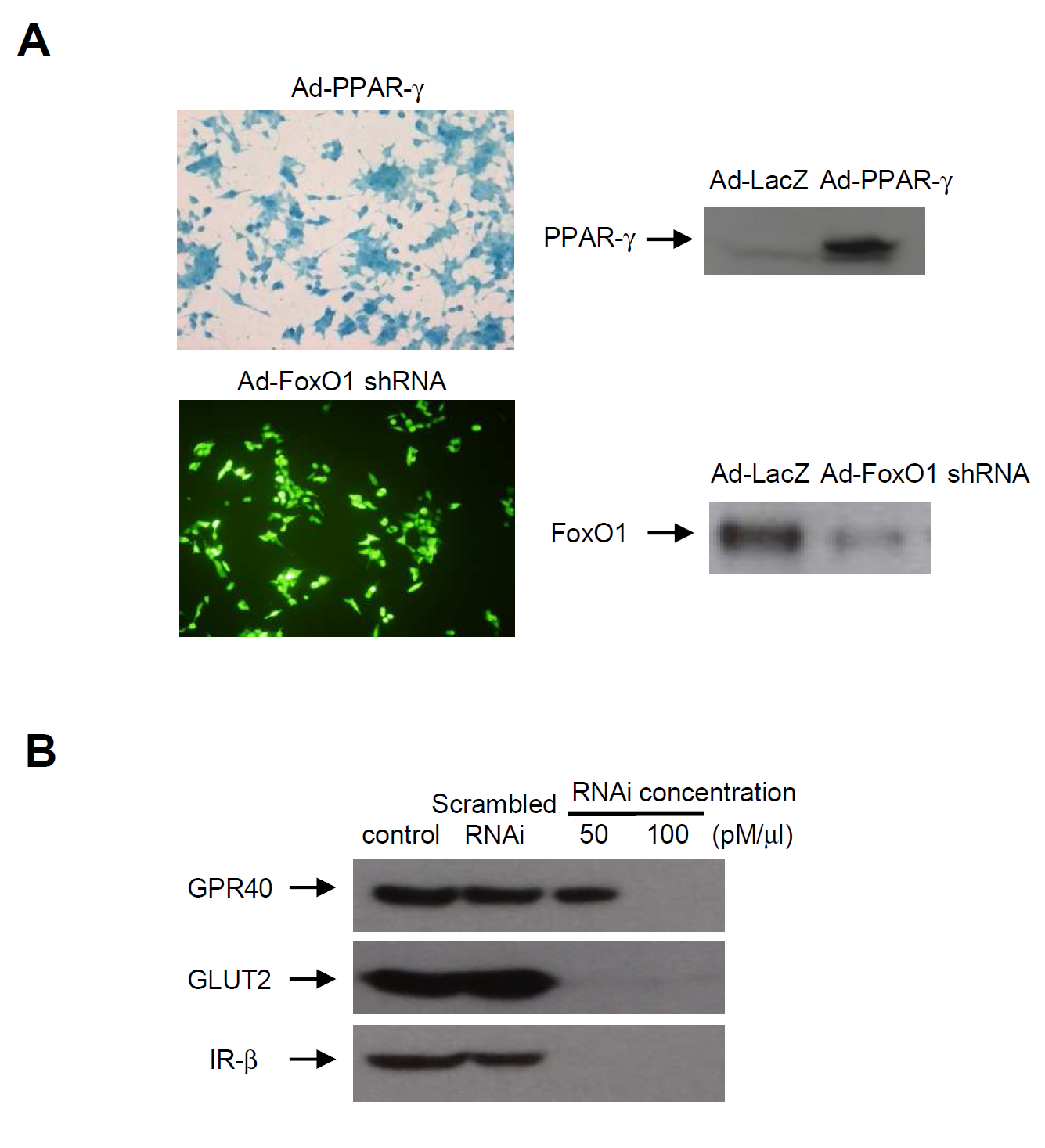

Supplement: Figure S1 — (A) Overexpression of PPAR- γ and suppression of FoxO1 in INS-1 cells. Efficiency of adenovirus was determined by X-gal staining, GFP expression, and Western blotting. (B) Inhibition of GPR40, GLUT2, and IR-β expression with RNAi transfection. As the RNAi concentrations increased, target protein expressions were suppressed with RNAi transfection. (TIF) [file pone.0050128.s001.tif]

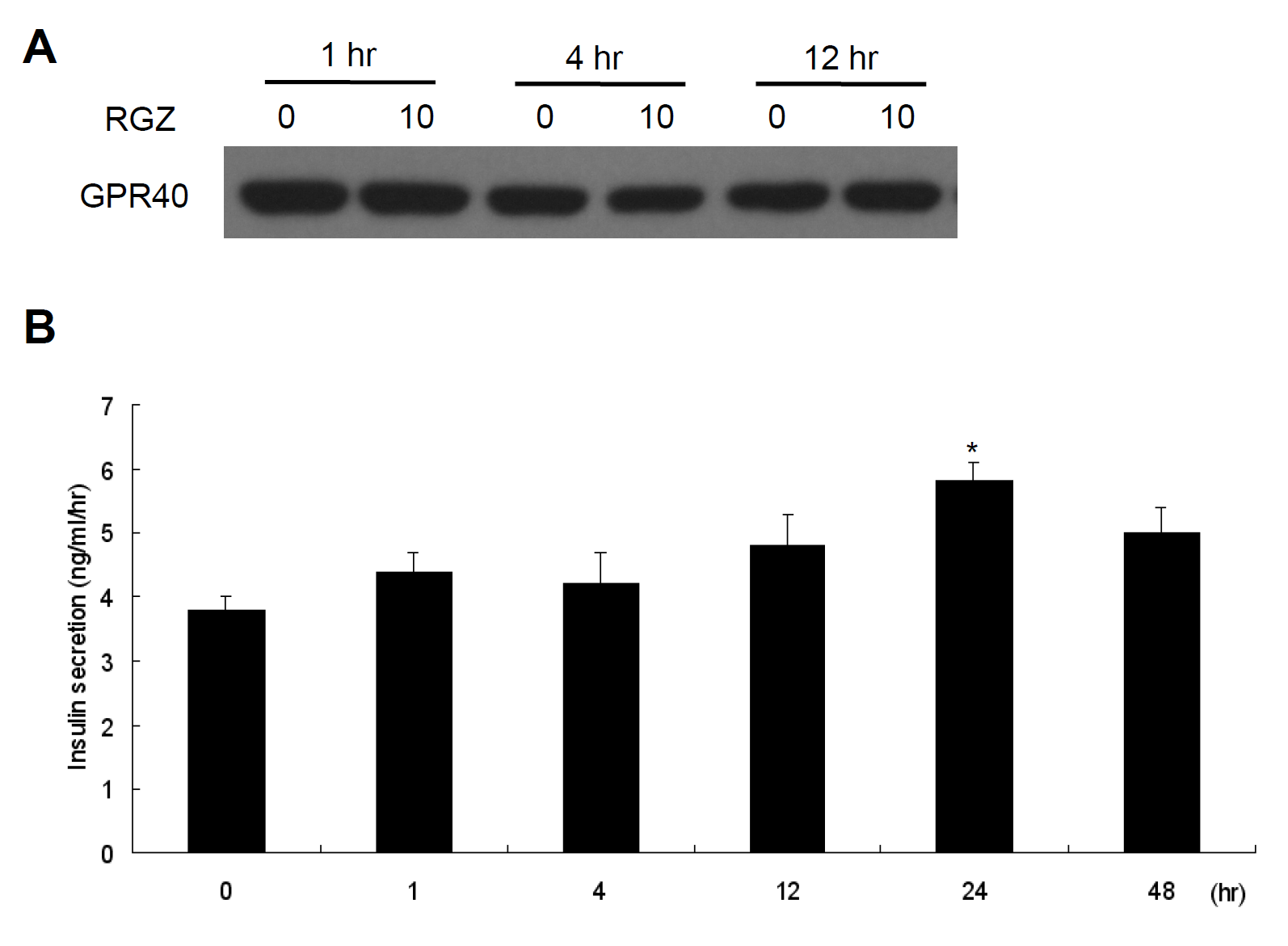

Supplement: Figure S2 — (A) Acute effect of rosiglitazone on GPR40 expression in INS-1 cells. (B) Acute effect of rosiglitazone on insulin secretion in INS-1 cells (n = 4, * P<0.01 vs. 0 hr). (TIF) [file pone.0050128.s002.tif]
